# Monitoring Pharmacological Treatment of Breast Cancer with MRI

**DOI:** 10.3390/cimb47100807

**Published:** 2025-10-01

**Authors:** Wiktoria Mytych, Magdalena Czarnecka-Czapczyńska, Dorota Bartusik-Aebisher, David Aebisher, Aleksandra Kawczyk-Krupka

**Affiliations:** 1 Department of Photomedicine and Physical Chemistry, Medical Faculty, Collegium Medicum, The Rzeszów University, 35-310 Rzeszów, Poland; 2 Department of Internal Diseases, Angiology and Physical Medicine, Center for Laser Diagnostics and Therapy, Medical University of Silesia, Batorego 15, 41-902 Bytom, Poland; magdalena.czarnecka921114@gmail.com (M.C.-C.); akawczyk@gmail.com (A.K.-K.); 3 Department of Biochemistry and General Chemistry, Medical Faculty, Collegium Medicum, The Rzeszów University, 35-310 Rzeszów, Poland; dbartusikaebisher@ur.edu.pl

**Keywords:** MRI, breast cancer, T_1_ and T_2_, treatment, diagnosis

## Abstract

Breast cancer is one of the major health threats to women worldwide; thus, a need has arisen to reduce the number of instances and deaths through new methods of diagnostic monitoring and treatment. The present review is the synthesis of the recent clinical studies and technological advances in the application of magnetic resonance imaging (MRI) to monitor the pharmacological treatment of breast cancer. The specific focus is on high-risk groups (carriers of BRCA mutations and recipients of neoadjuvant chemotherapy) and the use of novel MRI methods (dynamic contrast-enhanced (DCE) MRI, diffusion-weighted imaging (DWI), and radiomics tools). All the reviewed studies show that MRI is more sensitive (up to 95%) and specific than conventional imaging in detecting malignancy particularly in dense breast tissue. Moreover, MRI can be used to assess the response and residual disease in a tumor early and accurately for personalized treatment, de-escalate unneeded interventions, and maximize positive outcomes. AI-based radiomics combined with deep-learning models also expand the ability to predict the therapeutic response and molecular subtypes, and can mitigate the risk of overfitting models when using complex methods of modeling. Other developments are hybrid PET/MRI, image guidance during surgery, margin assessment intraoperatively, three-dimensional surgical templates, and the utilization of MRI in surgery planning and reducing reoperation. Although economic factors will always play a role, the diagnostic and prognostic accuracy and capability to aid in targeted treatment makes MRI a key tool for modern breast cancer. The growing complement of MRI and novel curative approaches indicate that breast cancer patients may experience better survival and recuperation, fewer recurrences, and a better quality of life.

## 1. Introduction

**Figure 1:**
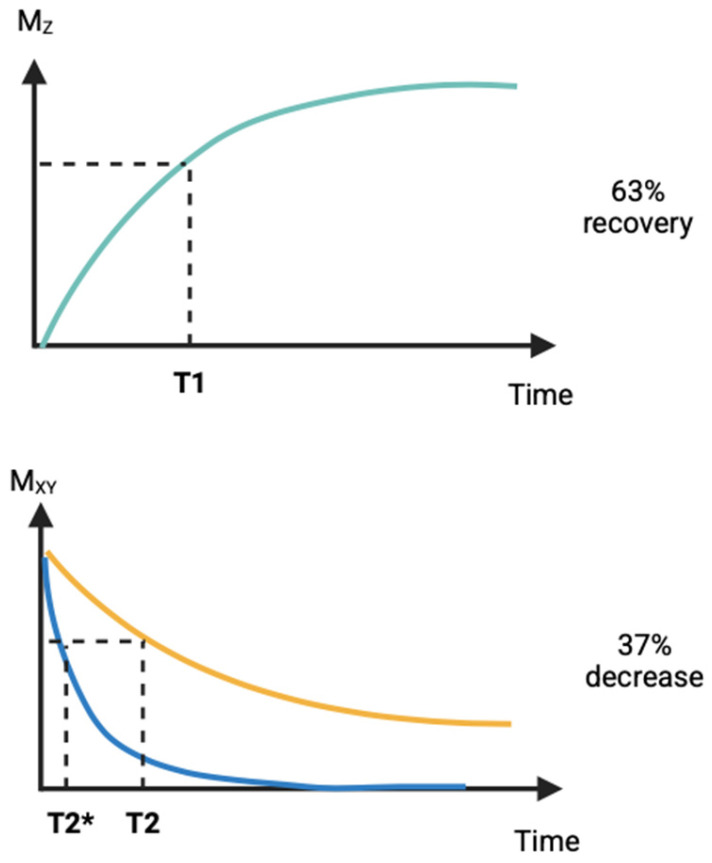
The image shows two basic processes of MRI relaxation: longitudinal relaxation (T_1_) and transverse relaxation (T_2_ and T_2_*). The two primary MRI relaxation processes that are visible in the image are longitudinal relaxation (T_1_) and transverse relaxation (T_2_ and T_2_*). Relaxation T_1_ is the recovery of magnetization in the *Z*-axis (perpendicular to the magnetic field) after an RF pulse. During the T_1_ period, the signal recovers about 63% of its initial value. This process, which is used to differentiate anatomical structures, depends on the molecular properties of the tissue, especially when contrast is added. The loss of spin coherence that causes the loss of magnetization in the transverse (XY) plane is referred to as “T_2_ relaxation”. During T_2_, the signal can drop by up to 37%. In addition, T_2_* considers the inhomogeneity of the magnetic field, which accelerates signal decay. The T_2_ and T_2_* parameters are particularly useful for detecting pathological changes.

One of the latest and most accurate diagnostic methods in the field of breast oncology is magnetic resonance imaging (MRI), the importance of which is growing over time not only in terms of detection and assessment of treatment, but also in terms of disease prognosis [1]. Monitoring the pharmacotherapeutic treatment of breast cancer involves using MRI to non-invasively measure responses to treatment at the morphological, functional, and molecular levels [2]. The method uses the nuclear magnetic resonance of hydrogen nuclei to obtain images with a high spatial and contrast resolution [3]. First, the patient is exposed to a strong homogeneous magnetic field, usually of 1.5–3 tesla, which will align the proton spins along the axis of the field. These are then excited by RF pulses on protons [4]. When switched off, protons are returned to equilibrium and emit a radio signal that is captured and converted into an image form. In magnetic resonance imaging, longitudinal (T_1_) and transverse (T_2_) relaxation times are recorded (Figure 1), and the dependence on these time values varies depending on the type of tissue, as well as the dependence on lesions [5]. Endogenous magnetic resonance imaging with contrast is based on the differences in T_1_ and T_2_ relaxation times between tissues due to the inherently different chemical composition, hydration, viscosity, and macromolecular content of these tissues [6]. This contrast makes it possible to distinguish between pathological and healthy tissues, including tumors, based on their physicochemical properties. In breast cancer, especially during the follow-up of pharmacotherapy, endogenous contrast may be insufficient to accurately determine the biological activity of the tumor, such as the rate of cell proliferation, angiogenesis, or metabolism [7]. As a result, exogenous contrast agents, which are usually gadolinium-based paramagnetic agents, are usually used. These agents alter the relaxation characteristics of aqueous protons, significantly increasing the differences in signals between healthy and pathological tissues, which allows for a more accurate tracking of changes in the therapeutic effect [8].

The use of breast cancer diagnosis and monitoring makes use of a complex set of imaging modalities that have their strengths and associated limitations considering their sensitivity, specificity, accessibility, cost, and clinical use. The screening gold standard is mammography which uses low-dose ionizing radiation to produce two-dimensional X-ray images of the breast but is sensitive to microcalcifications and early ductal carcinoma in situ (DCIS), with a sensitivity in the range of 70–80% in an average-density breast and specificity near 90% [9,10]. Nonetheless, genetic performance is even more impaired in thick breast tissue, due to overlapping glandular details that may obscure lesions, and, in this scenario, this may produce false negatives in up to 50% of younger women or those with fibroglandular density [11]. Digital breast tomosynthesis (DBT), a modernized mammography, solves this problem by offering three-dimensional reconstructions created during multiple low-dose measurements by increasing cancer documentation rates by 20–30% and lowering recall rates by 15%, but with increased radiation exposure and examination periods [12]. Although these improvements have taken place, there are cumulative risks of mammography due to the use of ionizing radiation, especially when carried out in high-risk groups such as carriers of the BRCA mutations [13]. Ultrasound is described as a complementary modality and is useful especially in differentiating between cystic and solid masses and applies with no radiation-based biopsies to guide biopsies. The sensitivity is approximately 60–85%, more so in dense breasts, where it is better, but less specific (70–80%), because it is operator-dependent and displays an overlap in detecting malignant and benign solid masses [14,15]. Automated breast ultrasound (ABUS) helps reduce inter-operator variability due to the use of standardized volumetric scans but also has limitations in deep-seated lesions or acoustic shadowing behind the breast and is costly, which limits its general application in resource-limited institutions [16]. Ultrasound monitors the decreases in tumor size with a predictable precision in pharmacological interventions such as neoadjuvant chemotherapy (NAC), but underestimates fibrotic residual disease, thereby enhancing overtreatment [17]. Positron emission tomography uses radiotracers such as ^18^F-fluorodeoxyglucose (FDG). In this technique, FDG is sensitive to axillary lymph node disease involvement at 85–95% and specific to viable tumor versus necrosis at 90% after treatment, aiding healthy organ growth. Positron emission tomography/computed tomography (PET/CT) that combines metabolic imaging using radiotracers and ^18^F-fluorodeoxyglucose (FDG) is best at identifying metastases (e.g., bone or liver) that are distant and may be limited by the field of view on MRI, and standardized uptake values (SUVs) can give a quantitative measure of the changes in targeted therapies such as anti-HER2 agents, in which there is a correlation between an appropriate pathologic complete response (pCR) achieved and a decrease in SUV levels (70–80%) [18,19]. Nevertheless, the high costs of PET/CT, radiation dose (comparable to 10–20 mammograms), lower resolution (4–5 mm), and false negativities in low-grade tumors (e.g., lobular carcinomas) limit PET/CT application in the primary screening test, as it is confirmed by biopsies. PET/CT has a better metabolic specificity and poorer soft-tissue distinction than MRI and is not the best when dealing with dense breasts or for evaluating multifocal disease [20,21]. Built-in magnetic resonance imaging (MRI), which uses magnetic fields and radiofrequency pulses, offers unprecedented soft-tissue contrast and functional imaging by sequences such as dynamic contrast-enhanced (DCE)-MRI and diffusion-weighted imaging (DWI), with some approaches reaching sensitivities of 95% and specificities of 85–90% in the highly sensitive screening of high-risk tumors and in NAC monitoring [22]. In contrast to the radiation exposure of mammography, MRI is not prone to any ionizing effect, thus being safer at repeated use and more sensitive when likened to ultrasound to quantify tumor vascularity and cellularity (ADC values), with pCR prediction accuracy in triple-negative subtypes ranging between 80–90%. Nevertheless, the time (30 to 60 min) needed for MRI to acquire a scan, the high cost, and the contraindications (e.g., pacemakers) contrast with the fast and cheap nature of mammographic runs [23,24]. MRI finds 1117 additional cancers in a dense breast in 1000 clinical screens than mammography does, with high costs of false-positive outcomes, resulting in unnecessary invasive procedures. PET/CT in metric systems serve as a complement of MRI in metastatic assessment, but the non-ionizing strength of MRI places it advantageously in longitudinal surveillance; however, it can also be used to great effect in concert with biomarkers [25]. Going further with the analysis, the sensitivities and specificities depend on the tumor subtype. In hormone-receptor-positive tumors, the sensitivity of MRI ranging 92–97% is better than PET/CT at 80–85% because of the reduced FDG avidity, whereas, in triple-negative tumors, the metabolic focus on PET/CT can detect signs of aggressive characteristics not visible to the morphological limits of ultrasound [26,27]. Barriers to clinical access highlight inequalities as the prevalence of mammography in low-resource settings, which highlights the need of MRI in centers—this is why developing nations experience higher mortality rates due to breast cancer [28]. In the monitoring of the treatment, mammography is able to follow the location of calcifications following radiotherapy, but, in the case of NAC, mammography does not work as the procedure tracks system fibrosis, although, in a real-time assessment, ultrasound is remarkably useful in the evaluation of volume (accuracy = 70%), and MRI is remarkably multiparametric as DCE and DWI predicts that the pCR is accurate with AUC = 0.85–0.95, and surpasses PET/CT with AUC = 0 [29,30]. Weaknesses are accumulated, specifically the radiation dose of mammography, the subjectiveness of the ultrasound, the tracer dependence of PET/CT, and the motion artifacts in MRI. Future approaches include the adoption of AI to improve particulars, e.g., 10–20% reductions in mammographic false positives with deep learning. Finally, the choice of modality depends on the level of risks to the patient, the breast density, and the clinical setting of the case. Multimodal approaches are optimal for minimizing the risks and uncertainties [31,32].

### 1.1. Contrast Mechanisms in Magnetic Resonance Imaging

**Figure 2:**
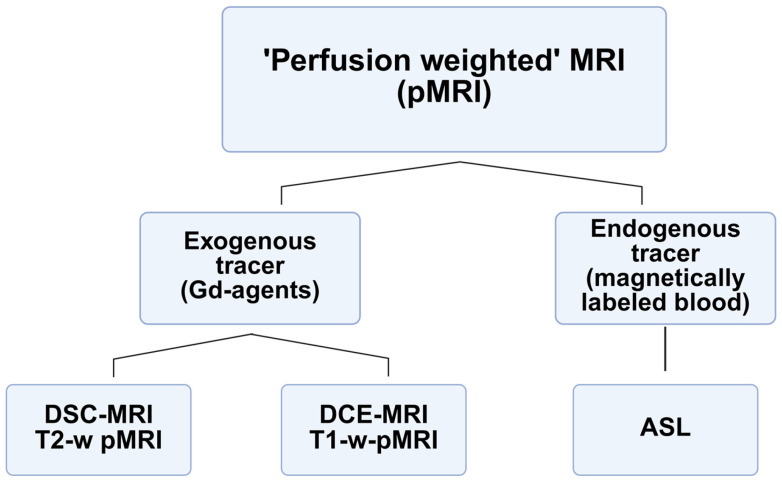
Schematic description of perfusion weighted MRI (pMRI) methods of perfusion measurement in the brain. In the upper row, the broad methodology is referenced as pMRI. The chart separates pMRI methodologies according to tracer environment: exogenous tracers (gadolinium-based agents) to the left, and endogenous tracers (magnetically labeled blood) to the right. DSC-MRI (T_2_-weighted) and DCE-MRI (T_1_-weighted) exogenous tracers, both represented by a typical brain perfusion map and respective quantitative variables, are plasma volume (Vp), blood flow (F), mean transit time (MTT/Tmax), transfer constant (Ktrans), and extravascular–extracellular volume fraction (Ve). The principle of cerebral blood flow (F) non-invasive measurement based on the endogenous tracer scheme, with arterial spin labeling (ASL) as an example, is presented as a perfusion map.

Contrast mechanisms in MRI are broadly divided into two categories: endogenous, which is based on the inherent physicochemical differences in tissues, and exogenous, which is based on exogenous contrast media (Figure 2). Endogenous contrast is caused by water and fat content, and cellular and macromolecular density. Tissues containing highly mobile water, such as areas of inflammatory swelling, may have long T_1_ and T_2_ values and, therefore, a low signal in T_1_-weighted scans and a high signal on T_2_-weighted scans [33,34]. Adipose tissues, on the other hand, have short T_1_ and T_2_ times due to their molecular composition and produce a high signal in T_1_ and a lower signal in T_2_. Tumor cellularity causes cells with limited extracellular space to be crowded, hindering the movement of water molecules and reducing the T_2_ and reducing the proton apparent diffusion rate (ADC) in diffusion-weighted magnetic resonance (DWI) [35,36]. Macromolecules (proteins) affect the rotational movements of water molecules, further altering the relaxation effects and distinguishing tissue signals [37,38].

Typical MRI abnormalities have been described in breast cancer, in the sense that T_1_ values are shorter in tumor tissue than in glandular tissue. T_2_ values can be variable, depending on the presence of edema, necrosis, or fibrosis, and ADC values are low in untreated tumor tissue and increase after successful treatment, secondary to cell death and the consequent expanded extracellular space [39,40]. Exogenous contrast is based on gadolinium chelates (Gd^3+^) and works through a large magnetic moment of Gd^3+^ ions which disrupts the local magnetic field of water protons, accelerating the relaxation T_1_ (and, less strongly, T_2_), so that there is a dramatic increase in the volume of the signal in which the contrast medium is concentrated, including vascularized tumors [41]. Gadolinium plays a major role in the assessment of perfusion and microcirculation in the monitoring of pharmacotherapy. The structural disruption, chaotic distribution of tumor vessels, and significantly increased permeability are characteristic of the supply of blood vessels prior to treatment, which allows a rapid influx of contrast and intense signal amplification [42]. The normalization of blood vessels has been shown to result in effective therapies, e.g., chemotherapy or antiangiogenic therapy, which are characterized by a delay in the speed of contrast inflow, a decrease in peak signal strength (SImax), and a delay in time to peak (TTP). In addition, treatments that lead to cell destruction enlarge the extracellular space, which can temporarily increase contrast uptake in the late stage [43,44]. Changes in time intensity graphs are particularly noticeable: type III breast cancer (rapid increase and decrease in signal) before treatment may become type II (plateau) or type I (slowly increasing with very little decrease) after treatment, reflecting a decline in angiogenesis [45].

### 1.2. MRI Sequences Used to Monitor Treatment Outcomes

Various MRI sequences are associated with a complete evaluation of the treatment effect, which affords information about tumor structure, function, and metabolic aspects to be obtained. T_1_-dependent values obtained prior to contrast administration can be used to analyze breast architecture, and find fatty foci (high signal), cysts (low signal), and fibrous elements [46]. Contrast-enhanced dynamic magnetic resonance imaging (DCE-MRI) is focused on contrast-dependent T_1_-mediated images, and the reduction in tumor gain and volume is assessed according to the RECIST (Response Evaluation Criteria in Solid Tumors) and mRECIST (modified RECIST) guidelines [47,48]. Triple-negative breast cancer (TNBC), TTP prolongation, and a decrease in SImax after the completion of two cycles of neoadjuvant chemotherapy (NAC) were significantly adjusted for pCRs [49]. T_2_-weighted images are used to assess the hydrated part of the tumor, swelling, necrosis, and fluid. After treatment, T_2_ signals may either temporarily increase due to inflammatory edema or decrease due to fibrosis, which is a late effect of treatment [50,51]. There are four morphological types of MRI responses, which are type I (smaller size), type II (fragmentation), type III (stabilization), and type IV (progression). Type II (fragmentation) had a favorable outcome when following hormone therapy, which was observed in 65 percent of patients in the aromatase inhibitor groups [52]. We have the contrast-enhanced dynamic magnetic resonance imaging (DCE-MRI) dynamic acquisition of a series of T_1_ images weighted at an interval of a few seconds after contrast and the processing of kinetic parameters such as SImax, TTP, leaching rate, leaching rate, and curve shape (type I, II, and III) [53,54]. Diffusion dependent imaging (DWI) using ADC maps quantifies the obstruction to the diffusion of water molecules that is reduced in cancer cells because they are surrounded by dense intricate cells and, therefore, have a low ADC. Successful treatment leads to cell lysis, the enlargement of the extracellular area, and ADCs [55,56]. Pulse-mapping T_1_ and T_2_ provides quantitative data on relaxation times in milliseconds, and identifies subtle molecular changes, including altered hydration or macromolecular composition, before changing the tumor size [57]. Magnetic resonance spectroscopy (MRS) analyzes metabolites such as choline, which is a marker of proliferation. A decrease in the choline peak during treatment means a poor level of metabolism and, consequently, a positive response [58].

### 1.3. Current Technological Status of MRI

Technological changes in the MRI surveillance of pharmacological therapy in breast cancer has become a foundation in precision oncology, defined by improved hardware, and refined imaging sequences with artificial intelligence (AI) adoption in the clinic and research environments [59]. Clinically, 1.5 T and 3 T MRI machines are predominant, which provide a high signal-to-noise ratio (SNR) mechanism and a high spatial resolution (1–2 mm) for the detailed visualization of breast tissue. DCE-MRI is reflected as a gold standard in tumor vascularity assessment tasks, including parameters such as the volume transfer constant (Ktras) and extravascular extracellular volume fraction (Ve), with a 90–95% sensitivity in early neoadjuvant chemotherapy (NAC) responses [60]. These studies show that DCE-MRI can forecast the pathologic complete response (pCR) with an area under the curve (AUC) of 0.85–0.91 following two NAC cycles, especially in triple-negative breast cancer (TNBC), in which vascular normalization is one of the most relevant treatment biomarkers [61]. Diffusion-weighted imaging (DWI) measures tumor cellularity as apparent diffusion coefficient (ADC) measurements, where rises in post-therapy cellularity (20–31% increases) signify the loci of cell lysis, and changes correspond to pCR in 80–85% of HER2-positive tumors [62,63]. Shortened MRI procedures have served clinical practice, with scan times of 10–15 min, similar diagnostic performance (sensitivity of 84–90%, and specificity of 80–85%), being economical in centers in clinical practice and enhancing patient tolerance [64]. The protocols emphasize significant sequences such as T_1_-weighted DCE and DWI and reduces unnecessary acquisitions, which have been confirmed in the study ECOG-ACRIN EA1141, which reached 87% sensitivity in the detection of invasive cancer in dense breasts. Specific breast coils can improve the SNR more than standard body coils by 30–50% and detect sub-centimeter lesions but process complications such as motion artifacts (in 5–10% of scans) require state-of-the-art motion-FORE algorithms [65]. Quantitative radiomics, the ability to predict molecular subtypes (e.g., luminal A vs. TNBC) with AUCs of 0.76–0.92 using convolutional neural networks (CNNs) with quantitative radiomics, offers a plethora of texture features such as the gray-level co-occurrence matrix (GLCM) on DCE-MRI, and can be improved by integration into quantitative radiomics [66]. Ultra-high-field (UHF) MRI systems (labeled 7 T or higher) extend the limits in research environments by providing molecular sensitivity at a level never seen before. These systems have spatial resolutions of less than 1 mm and an increased T_1_/T_2_ relaxivity, allowing magnetic resonance spectroscopy (MRS) to measure metabolites including choline (indicative of tumor growth) or lactate (indicative of hypoxia) with an 80–90% accuracy in preclinical TNBC models [67]. Another emerging technology, sodium (^23^Na) MRI, tracks intracellular sodium dynamics in response to PARP inhibitor therapy, which correlates well with apoptosis; yet, its clinical application is restricted by a low SNR and excessive scan times (45–60 min). Hybrid pet/MRI cameras combine metabolic and morphological data with 95% staging metastatic disease sensitivity in HER2-positive cancers; standardized uptake values (SUVs) and ADC are complementary to the treatment response [68]. For example, a 2023 study demonstrated that PET/MRI outperformed PET/CT in bone metastases detection (92% vs. 85% sensitivity) because of its better soft-tissue sensitivity [69]. There are ongoing technological issues such as the standardization of acquisition parameters across centers, which translates to a variation of 10–15% in ADC measurements as reported in a 2024 meta-analysis. This is reduced by bias field correction and harmonized (e.g., Breast Imaging-Reporting and Data System, BI-RADS), although multicenter trials are complicated due to inter-scanner differences [70]. Nephrogenic systemic fibrosis in patients with kidney failure has prompted the use of non-contrast techniques like T_1_/T_2_ mapping and arterial spin labeling (ASL) which cannot measure perfusion, but with reduced specificity (70–80%). Combined with AI designs, including deep-learning-based lesion localization, this brings down the intra-reader variance (kappa 0.8–0.9), as well as enhances the specificity by 10–15% in early experiments using LASSO [71,72] regression to refine radiomic features. The disadvantage is cost. Currently, the 3 T MRI scans’ cost makes them inaccessible to low-resource environments where mammography is predominant. Out-of-bore scanners contribute to the alleviation of claustrophobia (10–30% of patients), and the image quality can suffer because their field strengths are lower [73,74]. Nanoparticle-enhanced MRI in research with nanoparticles such as manganese ferrite increases the relaxivity of targeted imaging on pre-clinical models which demonstrated in TNBC models a 30% enhanced contrast [75]. Future techniques may use 11.7 T human scanners (to carry out state-of-the-art MRS or quantum sensing to offer metabolite findings) but regulatory challenges impede adoption [76,77]. 

### 1.4. Changes in MRI Parameters in Response to Therapies

Successful therapies cause well-known changes in MRI parameters because of biological response processes. In DWI, ADC is elevated due to cell rupture and the redistribution of water into the extracellular space. DCE-MRI SImax is reduced by normalizing blood vessels and reducing angiogenesis [78]. In DCE, there is an increased TTP which reflects a delay in contrast application by less porous vessels. Changes in T_2_ can be an increase (swelling or early phase) or a decrease (late phase of fibrosis). The choline peak of MRS is reduced, indicating reduced proliferation [79]. Magnetic resonance imaging can provide an early prognosis of non-response, allowing for the rapid adjustment of treatment. This is particularly useful in evaluating targeted (HER2-targeted, PARP-1 inhibitors) and immunological therapy where size-dependent measures may be inadequate [80,81]. Magnetic resonance imaging, along with radiomics and AI, predicts treatment outcomes before resizing, which is promising for therapy. Future trends include ultra-high-field magnetic resonance imaging (7 T) to improve the molecular sensitivity, and new contrast agents (e.g., manganese-based and receptor-specific), as well as coupling with genomic and proteomic data to support personalized medicine [82,83].

**Figure 3:**
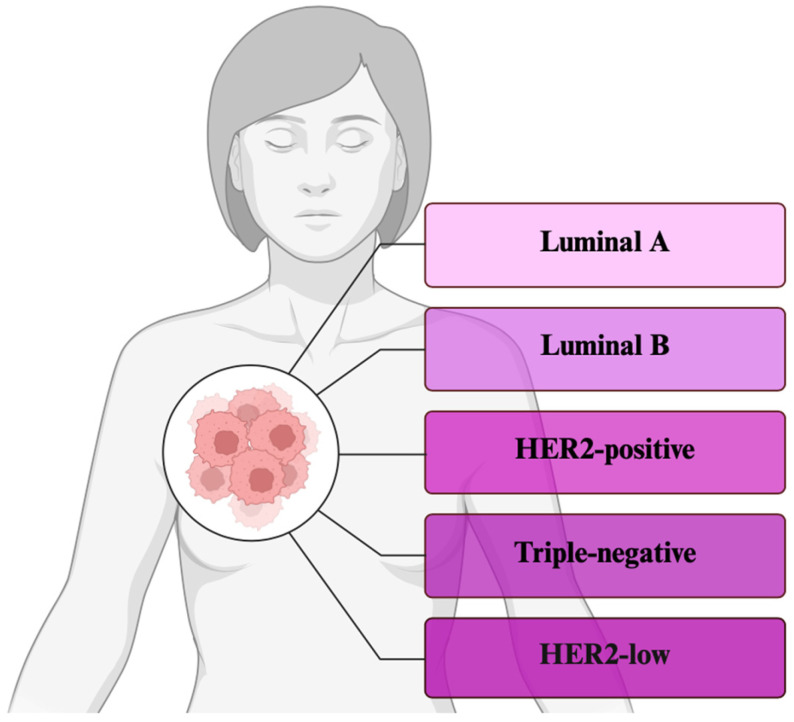
Subtypes of breast cancer.

Based on the expression of ER, PR, and HER2 hormone receptors, four molecular subtypes can be distinguished (Figure 3). Luminal A (ER+/PR+, HER2−, and low Ki-67 proliferation) has a low proliferation and, thus, a better prognosis [84]. High proliferation and poor prognosis (ER+/PR+, HER2±, and high proliferation) [85] are associated with Luminal B. HER2-positive is associated with aggressive progression and the overexpression of the HER2 receptor (ER-/PR-, and HER2+) [86]. Triple-negative (TNBC) does not express ER-, PR-, and HER2−, and, as a result, has an aggressive trajectory [87]. The HER2-low category or a group of cancers with a low level of HER2 expression has also been introduced [88].

**Figure 4:**
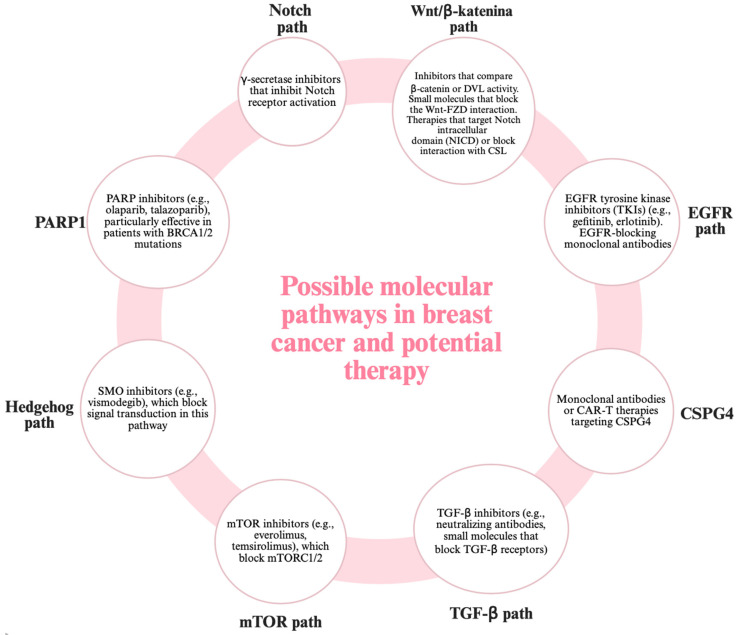
Possible molecular pathways in breast cancer. Activation by the Notch ligand leads to the transcription of genes that promote proliferation and differentiation [91]. The Wnt/β-catenin pathway, the Wnt ligand, binds to FZD and LRP5/6 receptors, activating DVL and β-catenin, which enters the nucleus and regulates gene expression [92]. Activation of the EGFR pathway stimulates the AKT and MAPK pathways, leading to drug resistance, tumor growth, and cell survival [93]. CSPG4 stabilizes interactions with the basement membrane of endothelial cells, promoting metastasis [94]. TGF-β binds to the receptor, activating SMAD2/3 → SMAD4, which promotes invasion and metastasis by IHH [95]. Activation of the mTOR pathway affects cell growth, protein synthesis, and tumor survival [96]. Activation of the PTCH receptor by the Hedgehog ligand leads to the activation of transcription factors GLI1, 2, and 3, which, in turn, induces SNAIL and JAG (invasion-promoting) genes [97]. PARP1 participates in the repair of single-stranded DNA (ssDNA) breaks, which promotes the survival of cancer cells, especially those with BRCA mutations [98].

Knowing the molecular mechanisms (Figure 4), the diagnosis of breast cancer has gained in specificity. The detection of the ctDNA of a circulating tumor using liquid biopsy has become one of the diagnostic tools [89]. There are also multi-gene tests that examine the expression of several genes. Next-generation NGS sequencing also allows for the identification of somatic and germline mutations [90].

## 2. Material and Methods

A literature review was conducted to reveal and evaluate the most recent research on the diagnostic and prognostic value of breast MRI. The review was conducted in relation to new peer-reviewed papers, such as clinical trials. Particular attention was paid to studies conducted on a population of high-risk patients: those who received neoadjuvant chemotherapy and those who were carriers of BRCA mutations. In terms of selection criteria, the emphasis is placed on studies using advanced MRI tools, including DCE-MRI and T_1_- and T_2_-dependent imaging (Table 1). The reason the studies were included was their importance in assessing tumor response, assessing residual disease, and evaluating the overall usefulness of MRI in the treatment of breast cancer. The main subjects of extraction were patient cohorts, clinically reported outcomes, imaging modalities, and the methodological quality of the studies. The results of the studies, which evaluate the combination of MRI with other forms of treatment, were also analyzed to determine possible synergistic treatments. This synthesis was aimed at presenting a full picture of the latest applications of MRI in breast cancer diagnosis and treatment planning. A literature review was conducted to identify and evaluate the most recent research on the diagnostic and prognostic value of breast MRI in monitoring pharmacological treatment. The search was performed across major databases, including PubMed, Scopus, Web of Science, and Embase, from 1998, to ensure inclusion of the latest advancements. The following keywords were used in various combinations: breast cancer, mammary carcinoma, breast neoplasm, MRI, magnetic resonance imaging, DCE-MRI, DWI, diffusion-weighted imaging, T_1_ mapping, T_2_ mapping, and pharmacological treatment. Boolean operators were applied to refine the search, and filters were set for peer-reviewed articles, clinical trials, and human studies published in English.

## 3. Results

The last decade has seen an intense development and increase in popularity of commercial and hormonal therapies for breast cancer, which, as a result of their specific functions, are used to specifically target cancer by engaging the mechanisms that govern tumor growth, survival, and metastasis. Compared to classic chemotherapy, these new approaches are mechanically differentiated and less toxic, and have new MRI kinetics.

MRI’s effectiveness in monitoring the pharmacological treatment of breast cancer has been greatly debated in literature, with convincing clinical research, meta-analyses, and review articles detailing the high sensitivity of the technology, with the limitations being highlighted in terms of the specificity, false positive outcomes, and meanings varying with the method of interpretation [99]. Standalone MRI, like with dynamic contrast-enhanced (DCE) sequences, can sense early morphology and functional changes, such as tumor volume shrinkage or the alteration in vascular permeability, with sensitivities of up to 90–95% in the case of neoadjuvant chemotherapy (NAC) to assess the pathologic complete response (pCR) [100]. As an example, in high-risk groups, such as carriers of BRCA mutations, MRI alone would identify residual disease with an 83–92% accuracy vs. mammography in dense breasts which detects occult malignancies in 16–20% of cases. Experts have, however, noted that, without complementary biomarkers such as circulating tumor DNA (ctDNA) and positron emission tomography (PET), MRI can be abused to inaccurately diagnose disease by its post-treatment effects of inflammation or fibrosis being interpreted as the response, resulting in at least 10–15% false positives on examinations and unnecessary biopsies [101,102]. Comprehensive research studies, like the I-SPY 2 trial, indicate that MRI has a prognostic role: the diffusion-weighted imaging (DWI) variation on the apparent diffusion coefficient (ADC) predicts pCR with AUC 0.85–0.91 with two cycles of NAC treatment, and these results diffuse throughout triple-negative breast cancer (TNBC) subtypes [103]. However, a meta-analysis of 11 studies on an abbreviated MRI indicates sensitivities of 84–95% but with specificities of 73–89% and false negatives of low-grade tumors where subtle metabolic changes are not identifiable without spectroscopy [104]. Problems such as inter-reader variability (kappa 0.6–0.8) involve the occurrence of perceptual errors (e.g., to spot small lesions) or bias (e.g., to interpret edema as progression), resulting in inaccuracy in 10–20% of cases [105]. In immunotherapy surveillance, uncombined with immune monitoring, pseudo-progression is recognized as T_2_ signal exceedances in 45–55% cases, whereas, without immune detection, pseudo-progression and actual progression cannot be differentiated in immunotherapy, which may delay therapy changes [106]. The errors are dependent on the subtype: MRI predicts the response to trastuzumab by an 87–93% sensitivity in HER2-positive cancers, but, in the luminal A group, the lower rate of proliferation causes underestimation [107]. One systematic review cites a systems issue of the contralateral breast evaluation MRI has, with a 3–5% occult cancer false positive rate; yet, the preclinical cost raises mastectomy by 8% (not showing survival advantages) [108]. The economic aspects make matters worse: the low cost acts as a barrier to access, and the long scan time will lower the throughput, further increasing the disparities in underserved regions [109]. Non-contrasts such as DWI can reduce gadolinium toxicity, but at the expense of specificity (70–80%), such as in studies where ADC alone fails to carry out vascular normalization in anti-angiogenic therapies [110,111]. The standalone MRI application use of personalized medicine is attenuated by the absence of integration with genomics; radiogenomics may promote better outcomes, whereas, at present, the protocol depends on a visual assessment with a 15–20% discordance to pathology [112]. Future trials such as POMB did not have any significant impact on survival due to preoperative MRI with more mastectomies and no lesser reoperations. Difficulties in dense breasts remain, with a 95% sensitivity of MRI that improves interval cancers but at a significant price of false positives [113]. In the future, AI can be used to improve the specificity to 90%, although, currently, standalone MRI can be used to offer high-sensitivity monitoring and it must be approached with care to prevent mistakes in diagnosis.

### 3.1. Targeted Therapies

#### 3.1.1. Anti-HER2 Therapy

**Figure 5:**
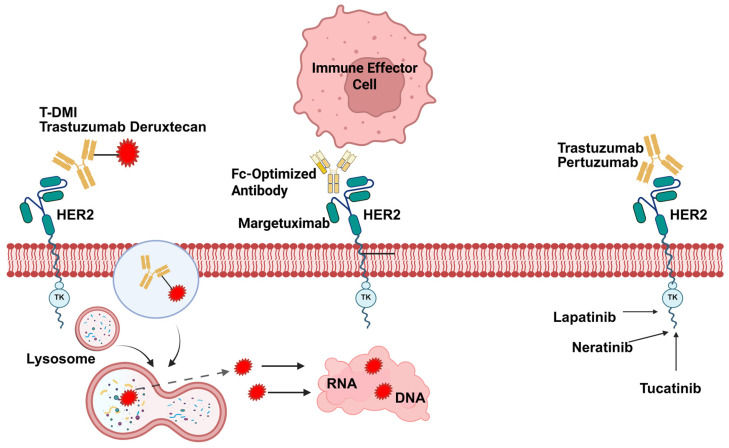
The figure shows localization of cell membrane receptors of HER2 and attachment of the various treatment compounds. On the left, the internalization of antibody-bound drugs (T-DM1, Trastuzumab Deruxtecan) occurs following their binding to the HER2 surface receptor and the subsequent release of RNA and DNA-damaging agents within the lysosome. The center panel illustrates antibody (Margetuximab) Fc engineered to both target HER2 and recruit immune effector cells. Intracellular targets on the right are HER2-targeted monoclonal antibodies (Trastuzumab, Pertuzumab) that block HER2 action and small-molecule tyrosine kinase inhibitors (Lapatinib, Neratinib, Tucatinib) that inhibit downstream signaling involving HER2-tyrosine kinase domain. The scheme unveils the existing strategies of combating HER2-driven breast cancer (direct inhibition, immunologic targeting, and intracellular conjugate worry).

The HER2 receptor (Figure 5) is a transmembrane tyrosine kinase that activates the PI3K/AKT/mTOR and RAS/RAF/MEK/ERK pathways and causes both proliferation and survival. The overexpression of HER2 in 15–20% of patients is associated with aggressive disease [114]. Drugs target HER2 Domain IV (monoclonal antibodies), Domain II (inhibition of HER2 dimerization), Domain II (antibody-drug conjugates), and tyrosine kinase (tyrosine kinase inhibitors) in the form of trastuzumab, pertuzumab, T-DM1, and T-DXd, and lapatinib or tucatinib, respectively [115].

**Figure 6:**
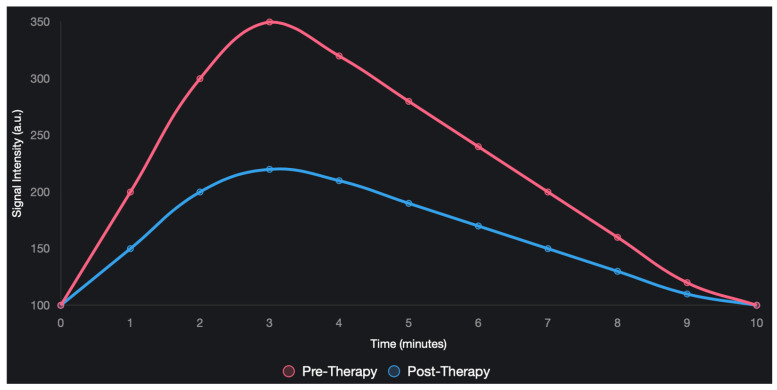
Pre-therapy (red) with a higher peak and faster wash-out, typical of aggressive tumor enhancement, and post-therapy (blue) with a lower peak and slower wash-out, suggesting reduced vascularity after anti-HER2 therapy.

Table 2 summarizes the studies on anti-HER2 therapies (Figure 6), detailing the patient populations, MRI methods, and main results. MRI techniques, including DCE-MRI, DWI/ADC, and PCMM-Net, were crucial in assessing the treatment response. Key findings include high AUC values (0.87–0.91) for predicting the pathological complete response (pCR) [116,117,118,119,120,121,122], significant ADC increases (20–31%) indicating the tumor response [117,120,123], and reductions in the tumor size, wash-in rate, SSmax, or E1 [116,117,120,121]. DCE-MRI and DWI/ADC were pivotal in evaluating early treatment efficacy, while PCMM-Net enhanced the pCR prediction accuracy [118,122]. CR on MRI strongly correlated with pCR, particularly in HER2− patients (87%) compared to HER2+ ones (53%) [119].

#### 3.1.2. PARP Inhibitors

**Figure 7:**
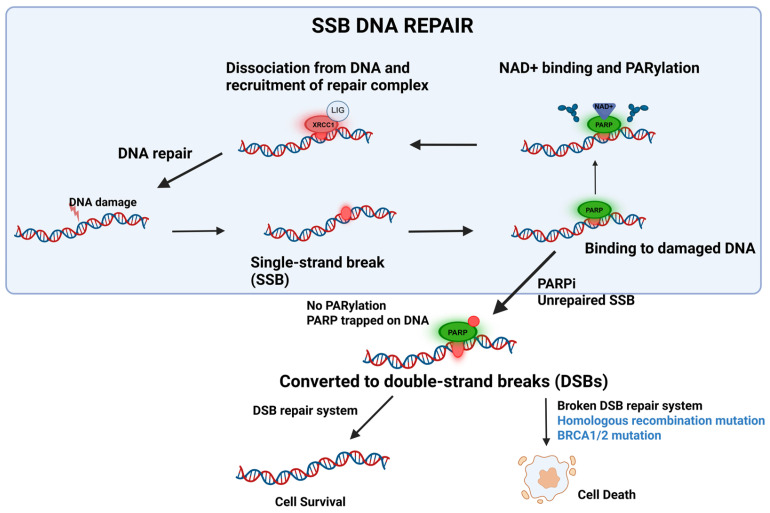
Schematic overview of the single-strand break (SSB) repair and the effect of PARP inhibition. Single-strand break (SSB) is caused by DNA damage during which PARP attaches to damaged DNA and is PARylated in an NAD+-dependent manner. This pathway calls on repair complex and re-establishes the integrity of DNA. The inhibition of PARP (PARPi) interferes with PARylation, as a result, locking PARP on the DNA, and SSBs are not repaired. Unrepaired SSBs become transformed into the double-strand breaks (DSBs). The survival of cells can be achieved through the DSB repair machinery; nevertheless, cells with dysfunctional homologous recombination, e.g., with mutated BRCA1/2, are unable to repair DSBs, causing cell death. The figure accentuates the pathway of normal SSB repair to the lethal effects of PARP inhibition in homologous recombination defective cells. In Table 2, we have summarized the information of PARP inhibitors use in therapy.

Drugs such as olaparib and talazoparib prevent poly(ADP-ribose) polymerase (PARP1/2) and stop the repair of single-stranded DNA (Figure 7). This results in the accumulation of DNA damage and the death of cells with a BRCA1/2 mutation (synthetic mortality) [124].

Table 3 summarizes the studies on PARP inhibitors in triple-negative breast cancer (TNBC) patients, focusing on the MRI methods and outcomes. MRI techniques, including ^23^MRI and DWI, were used to evaluate the treatment response. The key results include a 15–20% decrease in intratumoral sodium (Na^+^) levels, indicating cellular changes [125,126], and a 25% increase in ADC, reflecting the tumor response within 2 weeks [127]. Both ^23^MRI and DWI were effective in detecting the early treatment effects, with ADC increases and Na^+^ reductions serving as key biomarkers of PARP inhibitor efficacy [125,126,127].

#### 3.1.3. PI3K/AKT/mTOR Inhibitors

PIK3CA, PTEN loss, and AKT hyperactivation promote proliferation and resistance to endocrine oncotherapy [128]. Tumors in T1-DCE shrink to drugs such as alpelisib (PI3Ka inhibitor) or everolimus (m-TOR inhibitor), which increase ADC in DWI and deprive perfusion in the early phases of DCE [129].

### 3.2. Antiangiogenic Therapies

**Figure 8:**
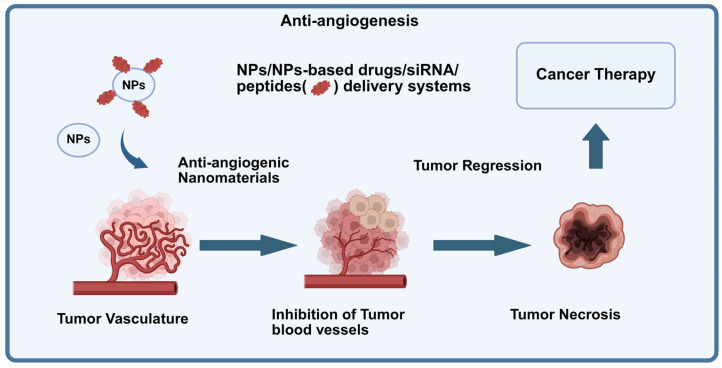
Examples of the application of nanomaterials (NPs) in anti-angiogenesis cancer therapy. The scheme represents nanoparticles (NPs) and NP-containing drug, siRNA, or peptide delivery to a tumor vasculature. The anti-angiogenic-based nanomaterial blocks formation and effects of tumor blood vessels, thus resulting in limited tumor blood supply. Vessel inhibition is followed by subsequent tumor regression and the development of tumor necrosis resulting in cancer therapy. The figure steps the sequence of nanodelivery system action to effective therapeutic outcome through anti-angiogenic mechanisms.

Angiogenesis inhibitors bevacizumab blocks VEGF-A and inhibits angiogenesis by normalizing blood vessels. In DCE-MRI, a decrease in Ktrans (contrast transfer rate) and SImax, and an increase in TTP over days to weeks are observed. The tumor volume can remain stable, which puts emphasis on the perfusion parameters [130] (Figure 8).

### 3.3. Hormone Therapies

#### 3.3.1. Aromatase Inhibitors (Letrozole, Anastrozole, and Exemestane)

Increasing androgens to estrogen, and, thereby, limiting ER+ cell stimulation, is inhibited by aromatase inhibitors [131]

#### 3.3.2. Selective Estrogen Receptor Modulators (SERMs)

Tamoxifen acts as an estrogen receptor antagonist in breast tissue, which means a progressive reduction in DCE enhancement, an increase in ADC after months, and a reduction in T_2_-associated perineoplastic edema [132].

#### 3.3.3. Selective Estrogen Receptor Degraders (SERDs)

Fulvestrant is another ER-binding agent that can degrade the ER, causing a sustained response, which is the loss of estrogen signaling [133]. On MRI, a reduction in tumor volume of at least 50% between T_1_-DCE and signal homogenization, and a decrease in the choline peak in MRS suggest that reduced proliferation occurs [134].

Table 4 summarizes the studies on hormone therapy in estrogen-receptor-positive (ER+) breast cancer patients, detailing the MRI methods and outcomes. MRI techniques, including MRS, T_2_, DCE, and DWI, were used to assess the treatment response. The key findings include a 45% reduction in choline levels with fulvestrant [133], Type II fragmentation in 55–65% of cases with aromatase inhibitors, letrozole, and anastrozole [134,135,136,137], and a 40% decrease in Buff with aromatase inhibitors [134]. Additionally, tamoxifen showed an increased ADC and reduced swelling [137], while letrozole led to a gain drop [137]. These MRI methods effectively detected early treatment-induced changes, with fragmentation, choline reduction, and an ADC increase as key biomarkers.

### 3.4. Immunotherapy

Immunotherapy, once considered a treatment during purely experimental research, has gained a foothold with respect to the treatment of certain subtypes of breast cancer, especially TNBC and immunogenic HER2-negative cancer [138]. It is designed to induce the body’s resistance and attack cancer cells. This has resulted in the need for new imaging techniques to detect atypical response patterns, including pseudo-progression (pseudo-tumor growth in response to the infiltration of immune cells within it) [139], hyperprogression (rapid tumor growth) [140], and changes in regional lymph nodes unassociated with metastasis [141,142]. Since morphological and functional properties are measured by magnetic resonance imaging, it can be concluded that this method would be suitable for identifying and distinguishing these phenomena. Checkpoint inhibitors: PD-1 inhibitors (e.g., pembrolizumab) and PD-L1 inhibitors (e.g., atezolizumab) block PD-L1 binding and PD-1 activation, thereby removing the blockage of the immune response [143]. This results in an exacerbation of cell inflammation and tumor immune infiltration, which, in MRI, can be observed through an increased enhancement in DCE-MRI contrast (depending on vascular permeability), an increase in the T_2_ signal (edema), and stable or slightly elevated ADC even despite the inflammatory infiltration of immune cells [144]. Antibody-drug (ADC) consignatures: Sacituzumab govitecan is an antibody conjugation against cytotoxic SN-38 and is cytotoxic only to cancer cells expressing the Trop-2 protein, not healthy ones. In MRI, early DCE enhancements decrease, ADC improves during initial cycles, and areas of necrosis show high T_2_ signals [145,146].

New molecules and vaccines: Peptide vaccines against HER2-low and MUC1 [147], a CSF1R inhibitor (altering the tumor microenvironment) [148], and STING agonists [149] are being investigated in clinical trials. MRI can reveal early swelling and an unusual altered DCE curve with both an enhancement and reduction in perfusion in various areas of the tumor.

Table 5 summarizes the studies on immunotherapy in various breast cancer populations [150]. The key findings include significant SImax reductions (35–40%) with pembrolizumab and atezolizumab [151,152,153,154,155,156,157,158,159,160], AUC values of 0.84–0.85 for predicting the pathological complete response (pCR) [154,161], and a 74–79% accuracy in response prediction with FMX-MRI [93]. Other results include a 40% reactive lymphadenopathy [151], increased T_2_ signal (45–55%) indicating edema or swelling [153,158], stable ADC in pseudoprogression [159], and 82% sensitivity for detecting pseudoprogression [157]. These MRI methods were critical for assessing immunotherapy efficacy and distinguishing pseudoprogression from true progression.

### 3.5. Radiomics

**Figure 9:**
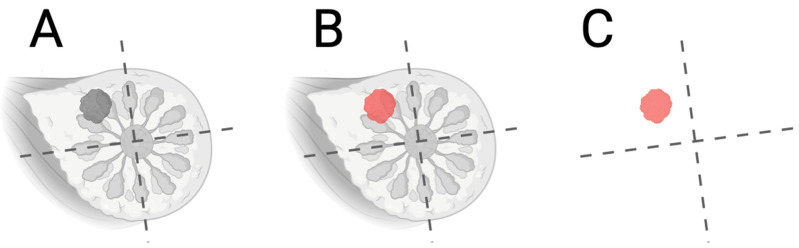
The diagram example illustrates the segmentation process of breast lesions by mapping the region of interest (ROI). (**A**) shows image of breast lesion surrounded by lobulated projections and burrs. (**B**) presents the segmentation image of the tumor from A, highlighting its irregular shape. (**C**) depicts the manual segmentation by drawing an ROI on the tumor in the same image as A, extracted in red using software.

Novel radiomics integrates many quantitative features automatically or semi-automatically extracted into medical images such as MRI, CT, or PET, and statistically analyzes and learns the results using artificial intelligence. Radiomics, unlike the traditional visual assessment, reveals subtle patterns of changes in texture, heterogeneity, shape, or contrast enhancement dynamics that can be taken as an expression of tumor biology in the presence of treatment [162]. In breast cancer, radiomics is used to predict neoadjuvant and targeted therapy outcomes and real-time treatment effects, and determine the biomarkers of drug sensitivity or resistance. For image acquisition and standardization, the main data are DCE-MRI, T_2_-weighted maps, and ADC on DWI sequences [163]. Resolution normalization, the correction of magnetic field heterogeneity, the standardization of signal intensity, and the segmentation of regions of interest (ROI) are all included in the standardization [164]. Contrarily, semi-automated 3D tumor segmentation in the initial stages of contrast enhancement contributed to the reproducibility of the results (Figure 9). The radiomic features are the shape (volume, sphericity, elongation, and compactness), intensity (mean, standard deviation, and histogram percentiles), texture (gray-level co-occurrence matrix—GLCM, gray-level run length matrix—GLRLM, and neighborhood grayscale difference matrix—NGTDM), and dynamic (leaching rate and washout rate, TTP, and AUC in DCE-MRI) [165]. Dimensionality reduction and modeling: With so many features, there is a risk of overfitting; in order to prevent this, a technique such as LASSO or PCA and cross-validation is used. Predictive algorithms include logistic regression, support vector machines (SVMs), random forests, or neural networks [166]. After a single course of treatment, radiomics reveals microstructural, perfusion changes (clearer uniformity of the DCE signal, lower directional variability of GLCM, and a slight increase in median ADC), which allows for the further personalization of treatment by continuing the treatment of non-responders until a response is achieved or the early discontinuation of treatment in those with a good response [167]. Radiomics can be integrated into genetic profiles (radiogenomics) by incorporating imaging features and genetic information about the tumor. The response to bevacizumab can be predicted in preclinical models in >90% of cases using combined DCE-MRI radiomics with VEGF/HIF-1alpha gene expression [168]. Multimodel models combine MRI, genomics, proteomics, and clinical data to provide a kind of digital twin of the patient [151,169,170,171].

Table 6 highlights that MRI detected 95.7% of cancers in dense breasts (17/17 invasive, and 5/6 DCIS) vs. DBT’s 39.1% [170], with the shortened MRI outperforming ABUS (17.4 vs. 4.2/1000) [172]. T1C-based CNNs (AUC 0.762–0.920) excelled in classifying TNBC and HER2-enriched subtypes [151,171]. DCE-MRI predicted the pCR (AUC 0.79–0.84) [173,174,175,176,177,178,179,180,181], with an early response linked to 87% HR- and 53% HR+ pCR [151]. Immunotherapy-related lymphadenopathy (44.4%) was unrelated to metastases [182]. Automated MRI segmentation improved the accuracy; EGFR therapy increased the ADC, and reduced the tumor size. The MRI biopsy showed a 95% pCR accuracy [183]. The abbreviated MRI maintained a high sensitivity (84.3%) with faster scans [174]. PET/MRI improved staging (81.9%) [175]; MRI detected 11% additional lesions, supporting radiotherapy omission (1% recurrence) [184]. H_DCE-MRI (AUC 0.963) and nomograms (AUC 0.88–0.936) enhanced lesion differentiation and TNBC/HR status prediction [176,177,178,179,180,181,185]. MRI’s high sensitivity, radiomics, and AI integration significantly enhance early detection, treatment monitoring, and diagnostic precision while reducing unnecessary interventions.

MRI is not the optimal resolution agent to monitor pharmacological therapy in breast cancer on its own because it presents a relatively low specificity, interpretive variability, and failure to resolve the articulation of the potentiation of molecular or systemic responses; in comparison, this feature exists with no supplementary usage [186,187]. Free MRI, specifically DCE-MRI and DWI, demonstrates value in the detection of changes early during therapy, with sensitivities where the prediction of pCR is 90–95% in the NAC setting, with sensitivities such as ADC increases of 20–31% post-therapy, and predicts survival in TNBC (hazard ratio 0.4) [188,189]. MRI in high-risk groups, including BRCA1/2 carriers, finds residual disease at 83–92/strongly predictive in high-density breasts, and identifies occult malignancy in 16–20/not detected by mammography or ultrasound. Standalone MRI in immunotherapy settings suffers pseudoprogression, where T_2_-weighted imaging LLDTs result in edema (signals rise in 45–55%) but lacks the ability to differentiate between this and actual tumor progression unless the imaging is accompanied by immune-related biomarkers, such as PD-L1 expression, leading to delayed treatment in not one-fifth of cases [190]. Substituting this, the pseudoprogression of TNBC with progressive disease on MRI is not easily detected in 15–25% of anti-PD-1 patients, making biopsy essential. The subtype-specific performance differs: in HER2-positive tumors, DCE-MRI has a sensitivity of 87–93% to predict the trastuzumab response, but, in low-proliferative luminal A tumors, sensitivity is reduced to 70–80% since subtle morphological changes are difficult to detect by this method [191,192]. Evidence of false positives includes a systematic review of preoperative MRI that reported a 26% false-positive rate in cases of extralateral breast assessment, raising mastectomy rates by 8% with no evident survival considerations, provoking the danger of overdiagnosis [193]. Standalone MRI is even more curtailed by economic and logistical hindrances. The cost and the length of the acquisition time (30–60 min) make it unavailable especially in low-resource environments where mammography is still a dominant technology [194]. Kappa (0.6–0.8) indicates inter-reader variability, which applies to diagnostic inconsistency, with perceptual errors for 5–10% of the interpretation, occurring in ECOG-ACRIN trials [105]. We use the negated gadolinium risk non-contrast MRI to mitigate the gadolinium risk, whereas it underestimates the effect on vascular responses in anti-angiogenic therapies, such as bevacizumab [195]. In the absence of biomarkers, like ctDNA or Ki-67, MRI cannot identify molecular changes, including PI3K/AKT pathway activation, resulting in a 15–20% discordance with pathological results in TNBC [196]. Clinical trials highlight the relative insignificance of MRI alone in the survival aspect since it did not detect any significant reduction in reoperations despite the rise of more mastectomies, implying that its high sensitivity must be matched against the risk of overtreatment. In dense breasts, the high sensitivity of MRI is 95%, and the interval cancer is minimized at the cost of 10–15% false positives; thus, the interpretation must be interpreted carefully [197,198]. Current advancements, e.g., AI-assisted lesion classification, could further raise the specificity to 90%, but, in its current form, standalone MRI cannot offer holistic molecular information, arguing in favor of multi-modal integration to maximize the delivery of therapeutic information.

## 4. Discussion

Magnetic resonance imaging plays a key role in detecting breast cancer in women and people with dense glandular breast structures, as well as in people at significant family risk (BRCA1/2 mutation carriers), in whom the screening capacity for mammography may be low. Magnetic resonance imaging allows the identification of bilateral, multifocal, and multicenter lesions as a method of planning the surgical and systemic treatment [199,200]. The monitoring of the response to treatment is also highly dependent on the method, especially at the time of neoadjuvant chemotherapy, as the effectiveness of the treatment can be evaluated at this time. DCE-MRI can provide useful prognostic information, such as angiogenesis and tumor biological activity, by monitoring the flow of contrast into the tumor [201,202]. The effectiveness of MRI diagnostics has been confirmed in many clinical trials. According to the American Cancer Society, breast cancer can be identified with a sensitivity level of 90–95% by MRI, better than by mammography (70–80%) or ultrasound (60–85%), especially in people with dense breast tissue [203,204]. 

The additional malignancies invisible on mammography were detectable by MRI in approximately 16–20 percent of patients. This can significantly change the line of treatment and increase its effectiveness [205,206]. The response to neoadjuvant chemotherapy is also potentially observed on MRI with a sensitivity of 83.92 and 88.95 upon the detection of residual tumor masses [207]. This allows for a very accurate assessment of tumor regression and the maximization of subsequent treatment. Multicenter studies, including I-SPY 2, show that DCE-MRI can reliably predict the response to treatment across several cycles of therapy, enabling the individualization of treatment and a reduction in side effects [208,209]. The MRI screening process significantly increases the early detection of cancer among BRCA1/2 carriers. This results in a very high five-year survival rate compared to patients monitored only by mammography [210,211]. The cost of a single examination is also higher, but economic analyses also show that the overall cost of treatment for MRI is lower, as appears to be the case in a subset of the population, because lesions can be detected early, after which some complications can be prevented, and less invasive surgical procedures may ultimately be required [212,213]. It is also important to note that MRI does not use ionizing radiation, and, therefore, it is a safe process to repeat when performing frequent examinations on the patient over an extended period. The dynamic development of technology and current clinical information lead to the recognition of breast MRI as the most effective diagnostic and prognostic intervention in the field of breast oncology [214]. The high level of precision in mapping tissue structure and function, the powerful use of functional imaging, and the growing importance of AI algorithms in MRI all contribute to making it the gold standard of diagnostic imaging. This supports a more personalized approach to breast cancer treatment and increased efficacy in women around the world [215,216]. Progress in recent years in imaging processes and technologies supporting breast cancer treatment has had a significant impact on the accuracy of diagnosis and the precise way of constructing surgery, as well as on the decision on the course of treatment. The determination of the location of the axillary lymph nodes (ALN) is one of the important research areas that can be carried out without cutting into the body using magnetic resonance imaging and state-of-the-art image analysis technology. Yu et al.’s project developed a radiomics method that uses magnetic resonance imaging and machine learning to predict the presence of metastases in ALN in people with early invasive breast cancer. The model, which included radiomic features and clinical and pathological information, achieved a high AUC of up to 0.93. The variability in radiomic characteristics after neoadjuvant chemotherapy was related to the characteristics of the tumor microenvironment, indicating that imaging markers are biologically established [217]. Current attempts to revisit margin-checking methods in breast-conserving surgery (BCS), which itself requires reoperation (in about a quarter of cases), have been carried out by researchers. Mobile magnetic resonance imaging and diffusion imaging using the ClearSightTM system showed a high sensitivity (80%) and specificity (84%) in margin control in people with DCIS and invasive cancer. In the events where surgeons were able to see the results of this system in real time, they could perform fewer re-surgeries. Reoperation rates can decrease by 83 and 50 percent, respectively, for invasive cancer and DCIS [218]. The relationship between preoperative MRI and postoperative survival and treatment outcomes is still widely debated. In a randomized trial in 524 patients, there was no significant benefit in overall survival or relapse-free survival with MRI. Nevertheless, it was associated with an 8% increase in the rate of mastectomies, with no effect on the number of reoperations [175]. The second POMB study compared the results after 10 years and found that MRI had only a statistically insignificant effect on survival. Nevertheless, in the subgroups at stages I–III, the MRI group performed better [113]. Lymph node evaluation after neoadjuvant treatment is necessary despite the limited accuracy of MRI. As demonstrated in the study by Reis and colleagues, although the size of the lymph nodes decreases rapidly at the end of therapy, magnetic resonance imaging, in some cases, resulted in a false estimate of the nodes, and the diffusion picture was the only one associated with the pathological condition of the nodes. Currently, MRI cannot be relied upon as a replacement for surgical lymph node assessment [219]. MRI projection mapping on the breast surface, which is also a recently developed technology, is helpful in locating the tumor before a surgical session. A study by Amano et al. showed that this system reduced the surgeon’s burden on visualizing the tumor because it precisely located the position and size of the tumor using MRI data while the patient was in a supine position. This contrasted with the conventional approach, which recorded significant differences [220]. Augmented-reality-based systems have also shown a similar performance to traditional ultrasound location, but with better ratings of surgeon satisfaction and convenience [221]. Moreover, 3D printing technology gains a lot of popularity when it is implemented and planned at BCS. To excise the lesions, the location of the tumor obtained by magnetic resonance imaging can be referred to the breast surface using 3D surgical guides (3DP-BSG), which allows for the precise location of the resection [222]. Similar results were obtained in DCIS, where 3DP-BSG allowed for the effective removal of the tumor with safe margins [223]. These promising results are seen with intraoperative radiation therapy (IORT) and preoperative light beam irradiation (PBI) consisting of a single fraction in follow-up care. The Japanese IORT study and the ABLATIVE study showed evidence of minimal toxicity, a stable quality of life, a satisfactory cosmetic outcome, and a low recurrence rate [224,225]. Two years later, PBI after oncoplastic reconstruction is also safe and effective, and gives beneficial aesthetic effects [226]. Nevertheless, in contrast to traditional radiomics and clinical–radiology models, a deep-learning (l) model that integrates MRI and clinical data, the PCMM-Net deep-learning model, has increased the precision of LVI prediction in the diagnosis of lymphatic vascular invasion (LVI), which can form the basis of a personalized treatment plan [227]. Ultimately, an economic evaluation of routine preoperative MRI at DCIS showed that, although the procedures are more expensive, the risk of reoperation was slightly reduced, and, thus, DCIS may prove cost-effective at an acceptable level of cost [228]. Studies on the use of letrozole in preoperative hormonal therapy in premenopausal patients with ER-positive DCIS have shown that it may result in a significant reduction in tumor volume and change in biological markers and may signal non-surgical management in a selected patient population [229]. Emerging imaging technologies have become very important in the treatment and diagnosis of breast cancer. They can make it possible to discover known lesions, as well as other disease sites that are often overlooked. Better results can be achieved by using magnetic resonance imaging instead of contrast-assisted mammography (CEM), which is readily available to patients. MRI and CEM assessments were performed together in a study that enrolled 59 women with invasive breast cancer prior to surgery. MRI identified 66/68 known malignant lesions (97%) and CEM 67 (99%). They also identified 41 new lesions: 6 using CEM alone, 23 using MRI alone, and 12 using both techniques. MRI detected all six additional malignant lesions, while CEM detected only one; the difference was not significant. The positive MRI predictive values were greater than CEM (23% and 8%, respectively). The conclusion is that both tests can effectively detect known lesions, and MRI may be more sensitive to finding other lesions [230]. However, PET scans are also the subject of many studies. The RESPONSE study was a substudy of the PHERGain study evaluating the effect of clinical and molecular effectors on the post-therapeutic observability of HER2-positive breast cancer on [18F] FDG-PET. Low PET activity (PET [-]) was correlated with smaller tumors, a lower grade, no node metastasis, and a lower risk of recurrence, and high activity (PET [+]) had more aggressive features in 500 patients. The genetic profile showed different HER2 and metabolic subtypes, indicating biodiversity in the disease and the adaptation of PET in the selection of the treatment [231]. In patients with metastatic breast cancer in which the HER288 expression was not found but the CEA expression, a novel 68-Ga-IMP288-pretargeted immuno-PET based on a bispecific anti-CEA antibody was evaluated. Immuno-PET showed greater sensitivity (94.7%) in defining metastases than conventional methods (CT, MRI, and ^18^F-FDG PET), especially in lymph nodes, liver, bone, and brain. The tumor uptake/tumor volume did not differ, but immuno-PET showed stronger tumor activity, indicating the high potential of this method in monitoring and evaluating the therapeutic response [232]. A hybrid PET/MRI approach can be used. Excluding not only sentinel lymph node biopsy, randomized trials have also shown that skipping axillary surgery in a selected group of patients with early breast cancer is neither less effective nor carries a greater risk of worse outcomes, although sentinel lymph node biopsy remains the preferred choice by most physicians. Therefore, a robust imaging method to identify axillary lymph node metastases can replace surgery. Hybrid [^18^F] FDG PET/MRI was shown to have a higher negative predictive value of 70.5% in 246 patients compared to 59.0% in PET alone and 41.0% in MRI alone in detecting macro metastases. Data indicate that PET/MRI can improve treatment planning and contribute to reducing the need for surgery [233]. The combination of the new nanodrugs and innovative therapies with MRI tracking is a paradigm shift in the treatment of breast cancer as it allows managers a specific assessment of the wash effects through superior contrast conditions, as well as through the upon-target delivery mechanisms [234]. The core principle is the synthesis of manganese ferrite (MnFe_2_O_4_) nanoparticles functionalized with myricetin, in which the morphology and the surface properties are optimally regulated through hydrothermal synthesis to ensure maximum stability and biocompatibility, enabling both T_1_/T_2_-weighted MRI-enhancement capabilities and tumor-suppression by antioxidant capabilities [235,236]. With nano sizes of 10–50 nm, these nanoparticles have enhanced the relativity (r1 > 5 mM^−1^s^−1^), which enables the timely detection of vascular changes in neoadjuvant active conditions, whereas the flavonoid structure of myricetin suppresses proliferation by 30–50% of TNBC models through ROS behavior, complementing the DWI features of MRI [146]. In addition to this, antibody–drug conjugates (ADCs), such as sacituzumab govitecan, which target Trop-2, can be linked to MRI by inducing necrosis, which is revealed as elevated T_2_ signals, and predicts pCR performance with a high accuracy in metastatic cases, with measures of DCE kinetics [237]. Olaparib, a PARP inhibitor in BRCA-mutated tumors, can be used in combination with several recommendations with MRI, including its standalone spectroscopy, and fine choline peak decreases, indicating metabolic shutdown, but the nanoparticle encapsulation increases authorization, reducing dispersion to off-query and enabling ^23^Na-MRI following treatment [238]. PEGylated liposomal everolimus (mTOR inhibitor) for prolonged circulation is emerging, with perfusion mapping with gadolinium-doped nanoparticles, associated with a 20–35% gain in reducing tumors in PIK3CA-mutated subtypes [239]. Speculatively, hybrid nanoplatforms of drug-carrying radioactive nanoparticles hybridized with ADCs via MnFe_2_O_4_ would permit theranostic use, with MRI monitoring the drug release in cancers caused by HER2, where therapy resistance due to reduced drug delivery may be averted by a 40–60% regulated dose. Problems such as biocompatibility and clearance are encountered, albeit preclinical models indicating less toxicity, and this allows for clinical trials wherein the biodistribution is quantified by MRI [240,241]. In immunotherapy, STING agonists encapsulated in polymeric nanoparticles amplify immune infiltration, detectable as T_2_ edema on MRI, enhancing response rates in TNBC by 50% when combined with checkpoint inhibitors [242,243]. Future speculations involve CRISPR-loaded nanocarriers for gene editing, monitored via functional MRI for epigenetic changes, revolutionizing precision oncology.

## 5. Future Perspectives

It can be observed that the application of MRI on the surgery of breast cancer, changing its pharmacological management, can raise state-of-the-art developments in the future, ignited by the technological breakthrough and cross-functional cooperation [244]. High-field MRI is expected to yield increased molecular sensitivity and spatial resolution, allowing the detection of subtle therapeutic responses at the cellular level, e.g., depending on the metabolite profile of an organ system as measured by magnetic resonance spectroscopy. Hybrid modes, such as PET/MRI, may combine a functional metabolic viewpoint with high-contrast anatomic imaging that may enhance the prognosis in high-risk groups such as triple-negative breast cancer patients [245]. The emergence of radiomics and deep-learning solutions based on the artificial intelligence concept has the promise of an automated response prediction, minimizing overfitting, and the further personalization of the response confirmation through the combination of imaging features and genomic data. New contrast agents, e.g., manganese-based particles or receptor-targeted nanoparticles, can reduce the risk in gadolinium, and increase the tumor–microenvironmental specificity [246,247,248]. Moreover, future trials must broadly be multicentric trials, including extrapolations on the I-SPY-series, which will confirm this use of MRI in adaptive treatment strategies, possibly un-escalating modalities and raises in survival rates [103,249]. Economic studies indicate that a reduction in costs due to shortened protocols and AI-assisted interpretations may widen access theoretically, leading to a precision oncology paradigm, in which MRI-guided monitoring would maximize results, decrease incidents, and even increase the quality of life of breast cancer patients worldwide [250].

### 5.1. Technological Requirements

Affordable imageries that ensure the proper and effective administration of MRI in the cellular treatment of breast cancer, specifically, the response to neoadjuvant treatment, are necessary. A standard MRI scanner would require a field strength of 1.5 T or 3 T with factored gains of 3 T imaging modes having a better signal-to-noise ratio and better resolution, but this demands parallel imaging modes and multi-channel radiofrequency (RF) coils to achieve the benefit of shorter acquisition times and image quality [251]. Bilateral breast coils that are dedicated are required to cover the breast tissue, position it, and image both breasts, covering all the way from the clavicle towards the inframammary fold. The system should be able to handle DCE sequences, which are exercises that call upon the use of contrast agents in the form of gadolinium even when there is an intravenous injection to analyze how the vascularity and improvement sequence of the tumor appears [252]. More functional sequences including diffusion weighted imaging (DWI) for the calculation of apparent diffusion coefficient (ADC) values are desired in order to be able to predict the response to treatment, especially for the hormone-receptor-positive subsets and triple-negative subtype. The main reasons for its restriction are the need for motion correction software during registration to reduce patient-motion-related artifacts and the requirement for fat suppression (either chemical shift or subtracted images) to increase lesion visibility [253]. There must also be post-processing capabilities and gadgets of post-processing, such as computer-aided detection (CAD), provided fat saturation is not conducted, and there must also be contrast enhancement as observed with delayed-phase T_1_-weighted imaging [254].

### 5.2. Clinical Protocols

Spanning the measurement of the tumor response, MRI in the clinical management of breast cancer treatment has supported clinical protocols that focus on applying a systematic methodology when monitoring the tumor response, usually during treatment with neoadjuvant systemic therapies. The pretreatment baseline MRI is paramount to determining the tumor size, extent, and features which will be compared to some post-treatment scans to generate the precise response category: complete response, partial response, and progression or stable disease based on the RECIST criteria [255,256,257]. The conventional guidelines include having the patients lie sideways, reducing movements, and bilateral imaging, which covers all breasts. The important order sequences consist of T_2_-weighted (bright fluid) sequences to detect edema and cysts and the multi-phase injected T1-order of order to detect the early post-contrast stage, and, on the other hand, the observation of delayed post-contrast phase [258]. Contrast-enhanced univariate MRI is standardized in staging, early response, and post-therapy imaging with a high sensitivity (40–90%) of revealing residual disease depending on the invasive lobular carcinoma, and triple-negative and HER-positive malignancies. Several DWI techniques are combined to monitor the litters of ADC and tumor volume, which are predictive of the pathologic complete response during the middle of the treatment [259]. Some imaging intervals have not yet reached a unanimous choice, but, in most cases, they come before treatment, during it (after 2–3 cycles), and after treatment; despite that, MRI is not regularly applied in axillary assessment in patients with node-negativity or after treatment without a prior baseline. The preparation of the patient involves a contraindication screening to contrast, and measures taken that do not include MRI soon after biopsy would influence the contrast patterns of enhancement [260]. This is used with mammography in high-risk surveillance, since it is used on BRCA mutation carriers, and there are guidelines that high-risk mammography be carried out in conjunction with MRI every year, as there is less yield in high-risk MRI in older patients (over 60–65) [261].

### 5.3. Standardization

It is important that standardization is carried out to make MRI interpretation more consistent and to enhance the reproducibility rates across the facilities. The American College of Radiology (ACR) also offers parameters that define the appropriateness of its protocols and parameters of the examination to ensure consistency in the implementation of the protocols, and these parameters require a bilateral contrast-enhanced sequence and post contrast imaging timing performances [262]. Shorter scans of 10–15 min with a high sensitivity (>90%) to detect the mass and measure size are becoming more commonly used in order to be cost-efficient and effective in the screening and monitoring process, and these need to be measured by the ACR standard of accreditation [263]. The Breast imaging Reporting and Data System (BI-RADS) has been adopted to report findings as the universal one, of which the patterns of enhancements and the measures of response are the tumor volume and the ADC changes. Diffusion MRI standardization aims at making b-values and ADC thresholds consistent across sites to forecast the treatment response and is nowadays being researched on contrast-free diffusion and AI-assisted analysis to reduce inter-observer variability [264]. The national recommendations, including the ones of the National Comprehensive Cancer Network (NCCN) and ACR, suggest incorporations of MRI with additional modalities whereby a complete response is to be evaluated through a scoring of the baseline and the absence of over-evaluation in the cases where no mass is enhanced [265], whereas international organizations such as the European Society of Breast Imaging (EUSOBI) advocate standardized protocols even in abbreviated and molecular imaging methods [266].

### 5.4. Training of Healthcare Professionals

Appropriate training is also necessary in order to see that MRI implementation has its radiologists, technologists, and supervising physicians effectively trained to allow appropriate acquisition, and interpretation, as well as reporting. In the case of the interpretation of the physicians, pre-qualifications are board certification in radiology or another equivalent, the completion of an accredited residency training program containing an MRI focus, or 200 h of continuing medical education (CME) in MRI, including breast-specific use, artifacts, safety, and instrumentation. They will also need to record the interpretation of 500 MRI cases within the last year and 36 months [267]. To be accredited, it is required that they complete 15 h of CME in MRI annually or semiannually with at least half coming under Category 1. To upgrade one’s ACR specifications related to the case-based training of 100 breast MRI interpretations, specialized courses, including those offered by the Society of Breast Imaging (SBI) or EUSOBI, offer such training, including such basics like enhancement patterns and the determination of responses [268]. Core training on breast imaging (MRI) is offered to radiology trainees. To technologists, though it is not explained by physician-oriented rules, the certification of the ARRT would usually require them to have MRI certification and continuous educational programs to be able to manage the positioning and sequence optimization, as well as patient safety [268]. Only multidisciplinary training can be carried out with oncologists so that the protocol can be in clinical demand, but advanced proficiency is emphasized in the hands-on fellowship.

## 6. Limitations of MRI

Although MRI is without any equivalent in breast cancer imaging, the technique suffers numerous limitations, which affect its clinical application, which include expensive operation, technical failure, and contraindications which are patient-specific. The cost of the scanning equipment, specialized coils, and contrast agents makes scans potentially very expensive, which makes them less available in resource-limited contexts, and may contribute to inequality in healthcare. The resolution and sensitivity, although better with soft tissues, can be degraded by motion artifacts or field inhomogeneities. Accessibility has also been an issue as MRI envisages facilities and trained personnel to carry out the imaging, and this restrains its incorporation in regular procedures in underserved areas. The problem of patient compatibility is critical: patients who have a cardiac pacemaker or implantable defibrillator are at risk of broken or overheating devices because of electronic interference, which means that they should be operated with MRI variants that are compatible with their devices or face other risks. Claustrophobia has been observed in up to 10–30% of patients which may necessitate sedation or the use of open-bore scanners that may compromise the quality of the images. Gadolinium-containing contrast agents, critical to dynamic contrast-enhanced MRI, are associated with risks of allergic and nephrogenic systemic fibrosis in patients with renal failure, where the retention of gadolinium may cause fibrotic complications; therefore, non-contrast methods such as diffusion-weighted imaging have become increasingly popular. Moreover, the long scan time and inability to coexist with some treatment regimes, due to the presence of metallic implants during the previous procedure, are other negative aspects upon which MRI attracts the selection of patients and the optimalization of the protocol to reduce the adverse effects of the stated setbacks.

## 7. Conclusions

MRI has emerged as useful in the diagnosis, monitoring, and treatment of breast cancer with a superior level of sensitivity and functional insight as compared to historic imaging equipment. New developments as pointed out in this review affirm the importance of MRI not only in diagnostics, but also in enhancing treatment modalities, especially in high-risk individuals and patients on neoadjuvant or targeted therapies. When the radiomic approach and the artificial intelligence approach are combined with functional MRI tools, e.g., DCE-MRI and DWI, the prognostication is considerably enhanced, and treatment planning is more individualized. A better nodal and metastatic evaluation is achieved by hybrid imaging mechanisms, such as PET/MRI, which minimizes the need to rely on invasive surgical interventions. Intraoperative MRI-guided assessment, 3D-printed surgical guides, and augmented reality interventions are innovative ways that could improve precision in surgery and minimize reoperation rates. Notably, preoperative MRI has not been demonstrated to uniformly benefit survival across all patient populations, but it has been recognized to benefit select subgroup populations and affect surgical management and local recurrence. Negative effects such as the cost and the risk of higher mastectomy rates should be balanced with the cost–benefits of early yet precise tumor detection and the inhibition of disease progression. Overall, the dynamic technological focus of MRI offers a more one-on-one environment, efficient and less intrusive in the management of breast cancer outcomes, helping to continue to increase results and the quality of life experienced by the patient. Further studying and interdisciplinary cooperation will play an important role in realizing the potential of MRI in breast oncology maximally. The recent developments in the field of breast cancer treatment monitoring by MRI are a manifestation of a potential shift toward more dynamic, integrative, and personalized solutions to both cancer diagnostics and prognostics, combined with the use of AI, imaging format, radiogenomics, and hybrid modalities. State-of-the-art AI-driven radiomics exploits multiparametric radiomics data with deep-learning models, whereby, after minimizing overfitting by least absolute shrinkage and selection operator regression, the data can be used to predict the molecular subtypes with AUCs of 0.76–0.92. Such models enhance the specificity by 10–15%, as shown in a 2025 study where CNNs identified lesions in dense breasts at a 90% correct rate, with only a small number of false-positive outcomes. The AI also automates segmentation, thus minimizing the inter-reader variability, and provides a real-time capability in predicting the response, including assessing ADC shifts post-NAC with a 85% accuracy after a single cycle. Advanced imaging regimes, including high-temporal-resolution DCE-MRI, and abbreviated MRI, make imaging more efficient and increase efficiency. Shortened protocols, as exemplified in the BRAID trial, shorten the scan time to 10 min with an 87–90% sensitivity, meaning that MRI can be used in high-density breast screening areas to complement mammography. High-temporal DCE images enhancement kinetics by resolving the sub-second, enhancing the pCR prediction in the TNBC by a factor of 20% over standard protocols. Non-contrast ones such as T_2_r mapping and ASL are considered gadolinium-free, with a sensitivity of around 80% in perfusion imaging; yet, the specificity is poor at 70–75%. Personalized therapy: Radiogenomics, which relates imaging characteristics to genomic characteristics such as VEGF mutations or BRCA mutations, can predict resistance in 70–80% of PIK3CA-mutated tumors. Hybrid PET/MRI systems offer metabolic and morphological information with a 95% sensitivity in detecting metastasis and outperforming PET/CT with 30% better staging in bone metastases. Nanoparticle-enhanced MRI, such as MnFe_2_O_4_, increase the relaxivity by 30% and allows the theranostic tracking of drug delivery, which is demonstrated in preclinical TNBC models. Trends: Future trends are in the application 7 T MRI in molecular imaging. Sensors based on quantum technology in the detection of sub-micromolar metabolites may become groundbreaking in early response testing. The cost and standardization challenges remain, but multicenter efforts and AI-driven harmonization is going to enable more widespread adoption and make MRI a key strategy in personalized, adaptive oncology.

## Figures and Tables

**Table 1:** Inclusion and exclusion criteria.

Inclusion Criteria	Exclusion Criteria
(1) Studies focusing on adult women with breast cancer undergoing pharmacological treatments, with particular emphasis on high-risk populations such as carriers of BRCA1/2 mutations and recipients of neoadjuvant chemotherapy; (2) Use of advanced MRI techniques, including dynamic contrast-enhanced MRI (DCE-MRI), diffusion-weighted imaging (DWI), T_1_/T_2_ mapping, or radiomics; (3) Evaluation of MRI for tumor response, residual disease assessment, or prognostic utility; (4) Original research articles, clinical trials, or meta-analyses with clear methodological reporting; (5) Sample sizes of at least 20 patients for robustness.	(1) Non-human (e.g., animal or in vitro) studies; (2) Studies without MRI as the primary imaging modality; (3) Publications in non-English language (4) Case reports, editorials, or reviews without primary data; (5) Studies lacking quantitative outcomes related to treatment monitoring; (6) Those with significant methodological flaws, such as inadequate blinding or high attrition rates (>20%).

**Table 2:** Anti-HER2 therapy.

Author, Year	Population and Therapy	MRI Method	Main Results
Liu Y., 2023 [116]	182 patients, NAT + anti-HER2	DCE-MRI	AUC 0.91 for pCR after 2 cycles; decrease in wash-in rate
Yang L., 2024 [117]	HER2+, EGFR/HER2	DWI/ADC + DCE	ADC up 31%; tumor reduction by 23%
Xu L., 2024 [118]	95 HER2+ patients, trastuzumab + pertuzumab	PCMM-Net	AUC 0.89 after 1 cycle
van der Voort A., 2024 [119]	HER2+/HER2−, NAC + targeted	DCE-MRI	CR on MRI: pCR at 87% (HER2−), 53% (HER2+)
Zhang X., 2024 [120]	HER2+, T-DM1	DWI + DCE	ADC up 28%; decrease in SSmax
Mercoglianno MF., 2023 [121]	HER2+, tucatinib	DCE-MRI	E1 drop by 35% after 3 cycles
Kou L., 2023 [122]	HER2+, T-DXd	PCMM-Net	AUC 0.87 for pCR
Li J., 2023 [123]	HER2+, lapatinib	DWI	ADC up 20%

**Table 3:** PARP inhibitors.

Author, Year	Population and Therapy	MRI Method	Main Results
James AD., 2022 [125]	TNBC, PARP inh.	^23^MRI + DWI	Decrease Na^+^ by 20%; ADC growth
Park S., 2024 [126]	TNBC, olaparib	^23^MRI	Na^+^ decreased by 15% in 4 weeks
Telli ML., 2024 [127]	TNBC, talazoparib	DWI	ADC up 25% in 2 weeks

**Table 4:** Hormone therapy.

Author, Year	Population and Therapy	MRI Method	Main Results
Nathan MR., 2017 [133]	ER+, fulvestrant	MRS	Decrease in choline by 45%
Yankeelov TE., 2006 [134]	ER+, aromatase inhibitors	T2 + DCE	Type II fragmentation in 65%; buff decrease by 40%
Reis J., 2021 [135]	ER+, Letrozole	T2 + DCE	60% fragmentation; gain drop
Zhai G., 2013 [136]	ER+, Tamoxifen	DWI + T2	ADC increase; decrease in swelling
Khanyile R., 2025 [137]	Luminal A, Anastrozole	T2	55% fragmentation

**Table 5:** Immunotherapy.

Author, Year	Population and Therapy	MRI Method	Main Results
Zou J., 2023 [150]	TNBC, NAC + immuno	DCE-MRI	Decrease in SImax by 40%
Jacob S., 2025 [151]	HER2−, pembrolizumab	MRI nodes	40% reactive lymphadenopathy
Ravi H., 2023 [152]	Metastatic Cancer, Irinotecan + Immuno	FMX-MRI	74–79% accuracy in predicting responses
He J., 2024 [153]	HER2−, atezolizumab	T2	Increase in signal in 55% (edema)
Chen Y., 2025 [154]	TNBC, pembrolizumab	DCE radiomics	AUC 0.85 for pCR
Arora A., 2025 [155]	TNBC, pembrolizumab + chemo	DCE-MRI	SImax decrease by 35%
Hu Y., 2025 [156]	HER2-low vaccine	T2 + DCE	Swelling in T2 in 50%
Kim N., 2022 [157]	TNBC, atezolizumab	Radiomics	Sensitivity 82% for pseudoprogression
Zhang W., 2025 [158]	TNBC, CSF1R inh.	T2	Signal increase by 45%
Liao D., 2023 [159]	HER2−, pembrolizumab	DWI	Stable ADC in pseudoprogression
Zhang X., 2024 [160]	HER2-low vaccine	Radiomics	AUC 0.84 for pCR
Panthi B., 2023 [161]	TNBC, atezolizumab + chemo	DCE	Decrease in SImax by 40%

**Table 6:** Other clinical findings obtained by magnetic resonance imaging.

Authors	Number of Patients	Methods	Results
Comstock CE. et al. [170]	1444	Comparison of the effectiveness of truncated breast MRI with digital breast tomosynthesis (DBT) in women with dense breasts.	- Magnetic resonance imaging detected 17/17 invasive cancers and 5/6 DCIS; - DBT detected 7/17 invasive cancers and 2/6 DCIS; - Sensitivity: MRI 95.7%, DBT 39.1%; - Specificity: MRI 86.7%, DBT 97.4%; - Additional tests: MRI 7.5%, DBT 10.1%; - PPV: MRI 19.6%, DBT 31%; - Invasive cancer detection: MRI 11.8/1000, DBT 4.8/1000.
Yin H. et al. [171]	136	To evaluate the efficacy of CNNs based on different MRI sequences in determining the molecular subtypes of breast cancer.	- AUC for T1C models: 0.762–0.920; - AUC for ADC models: 0.686–0.851; - AUC for T2W models: 0.639–0.697; - T1C models are the best in terms of performance; - Better results for triple-negative and HER2-enriched subtypes than for luminal A and B.
Jacob S. et al. [151]	43	To determine MRI patterns associated with neoadjuvant immunochemotherapy response in patients with HER2-negative breast cancer.	- 44.4% of patients in the immunotherapy group had lymphadenopathy compared to 6.3% in the control group (*p* = 0.014); - Increased lymphadenopathy did not correlate with the presence of lymph node metastases; - Increased lymphadenopathy despite tumor shrinkage; - 11/12 of patients with enlarged nodes had negative pathological results in the nodes.
Gilbert FJ. et al. [172]	9361 (6305 with full analysis)	Comparison of the effectiveness of additional imaging techniques in detecting breast cancer in women with dense breasts and negative mammography.	- Cancer detection per 1000 tests: Shortened MRI: 17.4 (37 cases); ABUS: 4.2 (9 cases); Mammography with contrast: 19.2 (39 cases). - Detection rate of invasive cancer per 1000 tests: Shortened MRI: 15.0; ABUSE: 4.2; Mammography with contrast: 15.7. - Shortened MRI detected significantly more tumors than ABUS (*p* = 0.047); - Insignificant difference between short-frame magnetic resonance imaging and contrast mammography (*p* = 0.62); - Adverse events are rare, most often in the contrast mammography group.
Sutton EJ. et al. [183]	20	Comparison of accuracy of MRI biopsy with surgical excision in detecting pCR after NAC.	- pCR was found in 65% of patients (definition 1); - Negative MRI biopsy predictive value: 92.8%; - Accuracy: 95%; - Sensitivity: 85.8%; - Positive predictive value and specificity: 100%; - 1 false negative result.
Mann GB. et al. [184]	443	To evaluate whether patients with monofocal breast cancer can safely skip radiation therapy after breast-conserving surgery.	- 11% of patients had additional cancerous lesions detected by magnetic resonance imaging; - In the group without radiotherapy (201 patients), the 5-year rate of local recurrence was 1.0%; - QALY increased by 0.019; - Savings of approximately AUD 1980 per patient.
van der Voort A. et al. [182]	467 (235 hrs, 232 hrs+)	To assess whether it is possible to shorten the number of chemotherapy cycles in patients with an early radiological response.	- After 1–3 cycles: 36% of patients with HR and 29% of patients with HR+ had a complete radiological response; - After 1–9 cycles: 73% HR- and 59% HR+; - pCR in 87% of HR- and 53% of HR+ with CR MRI; - The most common side effects: neutropenia (37%), anaemia (16%), and diarrhoea (12%); - No treatment-related deaths.
Zeng Q. et al. [173]	142 patients with invasive breast cancer who underwent DCE-MRI before and after 2 NAT cycles	To compare radiomics and percentage change in tumor diameter (Diameter%) in DCE-MRI before and after two NAT cycles to predict response to treatment. Development of a tool for early, non-invasive prediction of NAT results.	Radiomics, particularly delta and early-NAT, are potential biomarkers for early, noninvasive prediction of NAT responses. Combining radiomics with clinical data increases prediction accuracy.
van Grinsven SEL. et al. [174]	518	Comparison of the diagnostic accuracy of the abbreviated MRI protocol with the full protocol in breast cancer screening in women with dense breast tissue.	The shortened MRI protocol has similar sensitivity (84.3% vs. 85.9%) and specificity (73.9% vs. 75.8%) to the full protocol, but half the read time (49.7 s vs. 96.4 s) and 70–80% shorter scan time.
Mota BS. et al. [175]	524	To evaluate the effect of preoperative magnetic resonance imaging on survival and surgical outcomes in patients qualified for breast-conserving surgery.	Magnetic resonance imaging increased the rate of mastectomy by 8% (8.3% vs. 0.4% in the control group), and did not affect the absence of local recurrence or overall survival; no difference in the rate of reoperations.
Jannusch K. et al. [176]	208	Evaluation of the effect of 18F-FDG PET/MRI on the change in therapeutic management and accuracy of UICC staging.	PET/MRI improved the accuracy of the UICC staging (81.9% vs. 62.5%), but changes in treatment occurred in only 2.4% of the patients. Conclusion: Conventional staging is sufficient to make treatment decisions.
Yin HL. et al. [177]	319	To evaluate the value of combined deep-learning-based MRI diagnostics in differentiating TNBC and BI-RADS 4 fibroadenoma and improving the diagnosis of radiologists.	The result of the AI combination reached an AUC of 0.944; SI support improved the AUC of juniors from ~0.83 to ~0.88 and the AUC of seniors from ~0.90–0.95 to ~0.92–0.98; and, artificial intelligence helps younger radiologists, in particular.
Wu Z. et al. [178]	140	Construction and validation of a radiomic nomogram model for differentiating DCISM from pure DCIS.	The nomogram model (AUC ~0.88–0.91) was superior to the clinical model (AUC ~0.67–0.72), with good calibration and clinical usability.
Liu Y. et al. [179]	140 (56 BI-RADS 4)	To determine whether H_DCE-MRI is superior to L_DCE-MRI in differentiating between benign and malignant BI-RADS 4 lesions.	H_DCE-MRI, especially the intrashift parameter Kep (AUC 0.963, sensitivity 100%, specificity 88.9%) was much better than L_DCE-MRI and radiologist assessment.
Qi X. et al. [180]	158 (38 TNBC)	Identification of the optimal DCE-MRI phase for TNBC diagnostics and development of a clinical–radiomic model for TNBC prediction.	Phase 7 DCE-MRI radiomics model: AUC 0.818/0.777; clinical radiomic model: AUC 0.936/0.886 (Training/Validation Set), which improved TNBC prediction
Li Y. et al. [181]	108 (TNBC)	Development of a DCE-MRI-based nomogram to predict pathologic complete response (pCR) after NAC in patients with TNBC.	Nomogram with AUC 0.84 (training set) and 0.79 (validation set); independent predictors: tumor volume, time to peak (TTP), and androgen receptor (AR) status.
Sang L. et al. [185]	98 (68 trainings + 30 validations)	Development of a radiomic-based nomogram with T2WI, ADC, and DCE-MRI to predict HR status in HER2+ breast cancer.	The best model combining features from three sequences: AUC 0.797 (training), and 0.75 (validation). Nomogram with radiomics and perineoplastic edema: AUC 0.815 (training), and 0.805 (validation).
Verburg E. et al. [128]	4783 women, 525 BI-RADS 3–5 lesions	To evaluate the potential to reduce biopsies and false positives in BI-RADS 3 and 4 Using multiparametric MRI CAD.	Ridge regression model, extraction of 49 features (full model) and 39 features (abbreviated protocol), and 10-fold crossover validation.

## Data Availability

No new data were created or analyzed in this study. Data sharing is not applicable to this article.

## Abbreviations

The following abbreviations are used in this manuscript:

MRI	Magnetic resonance
TNBC	Triplet-negative breast cancer
ALNs	Axillary lymph nodes
DCE	Dynamic contrast imaging
BCS	Breast-conserving surgery
IORT	Intraoperative radiotherapy
PBI	Partial-beam irradiation
LVI	Lymphatic vessel invasion
CEM	Contrast-enhanced mammography
